# Cutaneous and conjunctival habronemosis in horses treated at the Veterinary Hospital of the Santa Catarina State University, Brazil

**DOI:** 10.1590/S1984-29612024049

**Published:** 2024-08-21

**Authors:** Larissa Américo, Lúcia Padilha Cury Thomaz de Aquino, Anderson Barbosa de Moura, Gianlucca Simão Nadal Ribeiro, Joandes Henrique Fonteque, Andreas Lazaros Chryssafidis

**Affiliations:** 1 Laboratório de Parasitologia e Doenças Parasitárias – LAPAR, Departamento de Medicina Veterinária, Centro de Ciências Agroveterinárias, Universidade do Estado de Santa Catarina – UDESC, Lages, SC, Brasil; 2 Hospital Veterinário, Departamento de Medicina Veterinária, Centro de Ciências Agroveterinárias, Universidade do Estado de Santa Catarina – UDESC, Lages, SC, Brasil

**Keywords:** Equine health, dermatology, vector borne diseases, Saúde equina, dermatologia, doenças transmitidas por vetores

## Abstract

Habronemosis, also known as habronemiasis or habronematidosis, is a parasitic disease of equids caused by the larval stages of Habronematidae nematodes (*Habronema muscae*, *Habronema microstoma*, and *Draschia megastoma*) that are transmitted by muscid flies. The presence of aberrant infective larvae in the cutaneous and conjunctival tissues of these hosts results in granulomatous, exudative, and ulcerated lesions, also known as “summer sores.” In this study, we present a retrospective analysis of habronemosis cases in horses from the municipality of Lages, located on the Santa Catarina Plateau, a region with high altitudes and a temperate climate that differs from regions of Brazil where such parasitosis usually occurs. The equids were examined from 2008 to 2020 at the Veterinary Hospital of Santa Catarina State University. Sixteen patients were diagnosed and treated using macrocyclic lactones and wound cleaning. Most cases were recorded in autumn in horses (10/16, 62.5%) over 15 years of age (11/16, 68.8%), and the lesions were more frequently located in the conjunctiva (11/16, 68.8%). In scientific dissemination media, this is the first report of habronemosis on the Santa Catarina Plateau, Brazil. This information will contribute to the diagnosis, treatment and prevention of skin diseases in horses in this region.

## Resumo

Habronemose (sin. habronemiasis, habronematidose) é uma doença parasitária em equídeos, causada pelos estágios larvais dos nematoides Habronematidae (*Habronema muscae*, *Habronema microstomae Draschia megastoma*) que são transmitidos por moscas muscídeas. A presença de larvas infectantes aberrantes nos tecidos cutâneos e conjuntivais desses hospedeiros resulta em lesões granulomatosas, exsudativas e ulceradas, também conhecidas como “feridas de verão”. No presente estudo, apresenta-se uma análise retrospectiva de casos de habronemose em equinos do município de Lages, localizado no Planalto de Santa Catarina, região com elevadas altitudes e clima temperado, diferindo das regiões do Brasil nas quais normalmente ocorre tal parasitose. Os equinos foram examinados de 2008 a 2020 no Hospital Veterinário da Universidade do Estado de Santa Catarina. Dezesseis pacientes foram diagnosticados e tratados com lactonas macrocíclicas e limpeza de feridas. A maioria dos casos foi registrada no outono em cavalos-(10/16, 62,5%) com mais de 15 anos de idade (11/16, 68,8%), e as lesões localizaram-se mais frequentemente na conjuntiva (11/16, 68,8%). Em meios de divulgação científica, este é o primeiro relato de habronemose no Planalto de Santa Catarina, Brasil. Essas informações contribuirão para o diagnóstico, tratamento e prevenção de dermatopatias em equídeos desta região.

## Introduction

Habronemosis, also known as habronemiasis or habronematidosis, is a parasitic disease of horses and other equids such as donkeys, mules, and zebras (Traversa et al., 2008), caused by spirurid helminths of the genus *Habronema* (*Habronema muscae* and *Habronema microstoma*), and the closely related species *Draschia megatoma* (Barlaam et al., 2020). These helminths are primarily transmitted by muscids, with houseflies (*Musca domestica*) serving as the main vectors for *H. muscae* and stable flies (*Stomoxys calcitrans*) for *H. microstoma* (Schuster & Sivakumar, 2017).

In the life cycle of these helminths, infective nematode larvae are deposited by flies around the equid nostrils or mouth. These larvae are swallowed and reach the adult stage in the stomach, leading to gastric habronemosis, which is often subclinical (Pugh et al., 2014). However, when infective larvae are deposited on wounds, abraded skin or mucous membranes, they enter the tissue and do not complete their lifecycle. When the larvae persist at a cutaneous or mucous membrane level, they induce an intense inflammatory reaction, resulting in cutaneous or conjunctival habronemosis, commonly referred to as “summer sores”.

Habronemosis lesions cause aesthetic damage and, depending on their extent and location, can directly interfere with animal use in sports or work activities (Bianchi et al., 2016; Assis-Brasil et al., 2015). When the larvae are released into the eyes or periocular tissue, infected animals may present with conjunctivitis, blepharitis, dermatitis with photophobia and lacrimation (Gasthuys et al., 2004; Verhaar et al., 2018).

Habronemosis displays a seasonal pattern, with climatic conditions directly impacting the proliferation of vectors, leading to the emergence of lesions during periods of heightened fly activity, typically in spring and summer seasons (Littlewood, 1999). Lesions may spontaneously regress during cool weather in the winter months; however, some infected equids may experience annual recurrences (Traversa et al., 2007; Pugh et al., 2014; Verhaar et al., 2018) .

Multiple therapeutic approaches have been proposed for habronemosis, all of which aim to eliminate the parasite and reduce the lesion size and associated inflammation. Treatment involves killing existing larvae with macrocyclic lactones, with ivermectin and moxidectin being the main reported drugs used (Xiao et al., 1994; Verhaar et al., 2018; Barlaam et al., 2020). The combined use of anthelmintics, corticosteroids and/or surgical excision have been presented using individualized protocols according to the location and extension of the lesion (Pusterla et al., 2003; Down et al., 2009; Pugh et al., 2014; Verhaar et al., 2018; Devi et al., 2019; Tyrnenopoulou et al., 2019; Salant et al., 2021).

Despite the worldwide distribution of habronemosis, little is known about the occurrence of cutaneous and conjunctival forms of the disease, mainly because of diagnostic limitations (Barlaam et al., 2020). The clinical diagnosis of summer sores can be challenging since the granulomatous lesions can easily be confused with a range of equid skin diseases.

The detection of larvae by microscopic examination of deep scraping or histopathology confirms the diagnosis; however, these methods have very low sensitivity, as larvae tend to be few and may be necrotic in more chronic lesions (Gasthuys et al., 2004; Verhaar et al., 2018; Tyrnenopoulou et al., 2019; Barlaam et al., 2020); the presence of parasite in histological examination has been reported as being present in only 44% of habronemosis cases (Pusterla et al., 2003). A semi-nested PCR protocol has been used for detecting *Habronema* spp. in skin samples with cutaneous lesions (Traversa et al., 2007; Down et al., 2009; Verhaar et al., 2018), even though a false-negative result was reported using that assay (Salant et al., 2021).

Therefore, the aim of the study was to conduct a retrospective reporting of horses attended at the Veterinary Hospital of Santa Catarina State University from 2008 to 2020, focusing specifically on cases of cutaneous and conjunctival habronemosis. This information will effectively contribute to the expansion of the clinical diagnosis of skin diseases in horses in temperate climate regions, as well as the development of new research to seek new alternatives for preventing this parasite in the southern region of Brazil.

## Materials and Methods

### Study location

The study was carried out by analyzing the records of horses attended from 2008 to 2020, in the Veterinary Hospital of Santa Catarina State University, with the specific focus on skin disease, selecting cases of cutaneous and conjunctival habronemosis. The hospital is located in the municipality of Lages, on the Santa Catarina Plateau, which attends to animals within the entire region.

### Evaluation of records and selection criteria

The medical records of all horses treated during that period were reviewed for skin disease, with subsequent selection of cutaneous and/or conjunctival lesions related to habronemosis. The criteria used to select cases included clinical suspicion, appearance of lesions compatible with cutaneous and conjunctival habronemosis, success of specific treatment and results of histopathological examinations, if any. Information regarding age, gender, lesion location, date of attendance, and treatment was compiled and analyzed. Data were evaluated using descriptive analyses of the animal care records.

## Results

During the survey period, a total of sixteen horses were clinically diagnosed with cutaneous and/or conjunctival habronemosis. All animals underwent treatment with 1% ivermectin administered orally at intervals of 30 days, totaling two to four doses, depending on lesion regression and the manufacturer’s recommendations. Regarding the lesions, cleaning was performed with 10% degerming iodine once or twice a day, depending on the severity of the lesion, for seven days or until its total regression. One of the animals performed a histopathological examination of the lesion, which revealed the presence of an infiltrate of eosinophils, macrophages and marked diffuse epithelioid macrophages accompanied by nematode larvae, which are in the midst of foci of caseous necrosis with mineralization. This type of histological lesion is compatible with habronemosis. All animals were monitored throughout the therapy period until complete regression of the lesions was achieved. Data obtained from the affected horses are shown in Table 1 and detailed below.

**Table 1:** Horses diagnosed with cutaneous and/or conjunctival habronemosis at the Veterinary Hospital of the Santa Catarina State University, in the period from 2008 to 2020. The table contains individual data regarding age, gender, location of the lesion, season/month and year when the animals were attended, diagnosed and treated.

**Animal**	**Age**	**Gender**	**Lesion site**	**Season / month**	**Year**
1	10	F	Cutaneous	Fall / June	2008
2	8	M	Cutaneous / Conjunctival	Summer / March	2012
3	NR^*^	M	Conjunctival	Spring / November	2013
4	> 20	F	Conjunctival	Fall / March	2013
5	20	M	Conjunctival	Fall / March	2013
6	> 20	M	Conjunctival	Fall / May	2013
7	< 20	M	Cutaneous	Fall / April	2014
8	> 25	M	Conjunctival	Fall / May	2014
9	20	F	Conjunctival	Fall / May	2015
10	<20	F	Conjunctival	Summer / March	NR*
11	18	M	Cutaneous	Fall / April	NR*
12	12	M	Cutaneous	Fall / April	2016
13	18	M	Cutaneous	Fall / April	2018
14	11	M	Conjunctival	Fall / April	2018
15	25	F	Conjunctival	Summer / February	2019
16	16	F	Conjunctival	Summer / March	2020

* NR: not recorded.

The affected horses ranged in age from 8 to 25 years or older, with the majority being over 15 years of age (11/16, 68.75%). Horses were more frequently affected (10/16, 62.50%) than mare (6/16, 37.50%).

**Figure 1:**
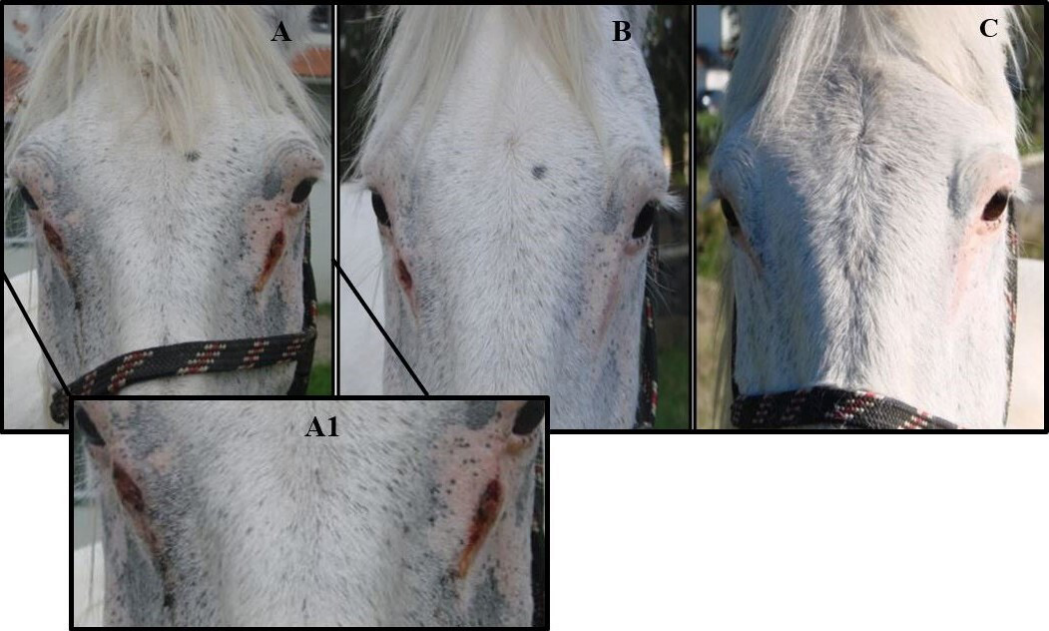
Horse with cutaneous habronemosis lesions, located in the medial corner of both eyes. The figure shows the regression of the lesions after treatment with Ivermectin. (A) First treatment; (A1) Enlarged image of the initial lesions; (B) During the treatment; (C) Complete healing after the end of treatment.

Of the 16 identified cases, conjunctival habronemosis was diagnosed in 10 horses (62.50%), while cutaneous habronemosis was detected in five animals (31.25%), and one horse (6.25%) presented both forms of the disease. However, all lesions identified as being caused by habronematid larvae were located on the faces of the horses, mostly under the medial canthus of the eyes (Figure 1).

Regarding the season and year of medical attendance and diagnosis, 11 cases (68.75%) occurred in the fall and four cases (25.00%) occurred in summer. Only one case (6.25%) was recorded in spring.

## Discussion

Despite the worldwide distribution of cutaneous and conjunctival habronemosis, significant gaps remain in our knowledge regarding its epidemiology, and the clinical and therapeutic aspects of this parasitosis, especially in areas with a temperate climate. In the present study, cutaneous and conjunctival habronemosis were diagnosed based on the clinical appearance of the lesions and their regression after anthelmintic treatment with ivermectin. The drug demonstrated to be highly effective (98–100%) in tests against gastric habronemosis (Xiao et al., 1994), as well as in a study carried out in Brazil, ivermectin demonstrated efficacies of 92.48% and 94.85% against two different *H. muscae* strains (Felippelli et al., 2015).

The determination of age prevalence in our survey was hampered by the lack of precise data in the medical records of some horses, but it was noticed that despite a wide variation in the age of the affected animals, none of them were less than eight years old. This fact is relevant in the current report, because the majority of owners did not use the animals for sport or breeding purposes, but rather for the purpose of using them as animals for transporting loads. In comparison to age, these findings are in line with those of two other reports, in which the youngest affected horses were eight and six years old (Verhaar et al., 2018; Salant et al., 2021).

In the present study, the lesions were more common in males than in females. This result corroborates the findings of two retrospective histopathological surveys conducted in the United States (Valentine, 2005) and Brazil (Bianchi et al., 2016), that reported a positive correlation between male horses and the occurrence of habronemosis.

Regarding the regions of the body most prone to summer wounds, these are the regions that are also the most susceptible to traumatic injuries linked to various factors, such as handling, sports or animal behavior. Cutaneous habronemosis has been reported most frequently on the limbs and medial corner of the eye (Pusterla et al., 2003; Down et al., 2009; Devi et al., 2019; Salant et al., 2021). Likewise, in non-cutaneous habronemosis, the most commonly diagnosed sites are the ocular conjunctiva and the urethral process of the penis (Tyrnenopoulou et al., 2019; Gasthuys et al., 2004; Pusterla et al., 2003).

Although generally not life-threatening, habronemotic lesions can lead to aesthetic and functional damage that may reduce the performance of equids in sports and work activities. Despite its importance, information on the distribution and prevalence of this disease worldwide is scarce, probably because of diagnostic limitations, which may cause an underestimation of the number of cases. Clinical diagnosis is challenging because of the similarity of habronemosis lesions to other horse skin diseases, such as cutaneous sarcoids, squamous cell carcinoma, mast cell tumors, and fungal and bacterial granulomas, which should be considered as differential diagnoses. Lesions of ocular habronemosis may resemble foreign bodies, neoplasia, onchocerciasis, and/or phycomycosis/botryomycosis (Pusterla et al., 2003; Pugh et al., 2014; Tyrnenopoulou et al., 2019).

Notably, the lesions found in the horses in this study were restricted to the face. On the Santa Catarina Plateau, it is still common to use horses for pulling carts, and almost all horses have been used for this purpose. The high proportion of conjunctival lesions observed may be due to the frequent use of blinders, which provide protection to flies, favoring their longer permanence in the ocular and periocular regions to which they are attracted by eye secretions. It is not possible to exclude the possibility that other lesions located in different body parts may have been confused with other skin diseases, as it was only possible to define a case as related to habronemosis, with clearly described and compatible lesions, and successful treatment using molecules related to such diseases.

The evaluation of seasonality showed that, except for one record in spring, all cases occurred in the summer and fall, mostly in March, April, and May (68,75%). This finding coincides with those obtained in a retrospective study based on the histopathological diagnosis of equine skin diseases conducted in southern Brazil, which identified a higher prevalence of cutaneous habronemosis from January to May (Assis-Brasil et al., 2015).

It is well known that habronemosis is prevalent in regions with tropical and subtropical climates because of the higher proliferation and activity of fly vectors. Nevertheless, summer sores have been also reported in colder climates in temperate regions of Belgium (Gasthuys et al., 2004), England (Down et al., 2009), the Netherlands (Verhaar et al., 2018), and Greece (Tyrnenopoulou et al., 2019).

These data were compared to the current retrospective study, as the climate in the Santa Catarina Plateau, is classified as temperate oceanic with constant humidity (Ricken et al., 2018). The region experiences periods of very low temperatures in winter, which can harm the proliferation of vectors. Seasonality helps to reduce cases of habronemosis in the winter and subsequent spring, as observed in this study, however it does not exclude the presence of positive cases in the summer and fall.

## Conclusion

No similar survey of cutaneous and conjunctival habronemosis was previously performed in the Santa Catarina Plateau. In the present study, it was demonstrated specifically that in the horses treated at the Veterinary Hospital of Santa Catarina State University, from 2008 to 2020, habronemosis occurred mostly on the face and eyes of male horses over 15 years old during Fall and Summer. Due to the small number of cases and animals, and the limitations of a retrospective study, it is not possible to extrapolate the conclusions (prevalence) to the entire equine population in the region. However, this report confirms the occurrence of cutaneous and conjunctival habronemosis in the locality, allowing clinicians to consider this disease in the differential diagnosis of dermatopathies in equids. This study contributes for advancing the knowledge on the epidemiology of habronemosis in Brazil, and highlights areas for further investigation.

## References

[B001] Assis-Brasil ND, Marcolongo-Pereira C, Stigger AL, Fiss L, Santos BL, Coelho ACB (2015). Equine dermatopathies in Southern Brazil: a study of 710 cases. Cienc Rural.

[B002] Barlaam A, Traversa D, Papini R, Giangaspero A (2020). Habronematidosis in equids: current status, advances, future challenges. Front Vet Sci.

[B003] Bianchi MV, Boos GS, Mello LS, Vargas TP, Sonne L, Driemeier D (2016). A retrospective evaluation of equine cutaneous lesions diagnosed in southern Brazil. Acta Sci Vet.

[B004] Devi CN, Borthakur SK, Patra G, Singh NS, Tolenkhomba TC, Ravindran R (2019). Incidence of cutaneous habronemosis in Manipuri ponies in India. Vet Parasitol Reg Stud Rep.

[B005] Down SS, Hughes I, Henson FMD (2009). Cutaneous habronemiasis in a 9-year-old Arab Gelding in the United Kingdom. Equine Vet Educ.

[B006] Felippelli G, Cruz BC, Gomes LVC, Lopes WDZ, Teixeira WFP, Maciel WG (2015). Susceptibility of helminth species from horses against different chemical compounds in Brazil. Vet Parasitol.

[B007] Gasthuys FMR, Van Heerden M, Vercruysse J (2004). Conjunctival habronemiosis in a horse in Belgium. Vet Rec.

[B008] Littlewood J (1999). Control of ectoparasites in horses. In Pract.

[B009] Pugh DG, Hu XP, Blagburn B (2014). Habronemiasis: biology, signs, and diagnosis, and treatment and prevention of the nematodes and vector flies. J Equine Vet Sci.

[B010] Pusterla N, Watson JL, Wilson WD, Affolter VK, Spier SJ (2003). Cutaneous and ocular habronemiasis in horses: 63 cases (1988-2002). J Am Vet Med Assoc.

[B011] Ricken P, Hess AF, Borsoi GA (2018). Relações Biométricas e Ambientais no Incremento Diamétrico de *Araucaria angustifolia* no Planalto Serrano Catarinense. Cienc Florest.

[B012] Salant H, Rojas A, Yardeny D, Brenner O, Schvartz G, Baneth G (2021). Cutaneous habronemosis in horses: first molecular characterization of *Habronema muscae* in Israel. Comp Immunol Microbiol Infect Dis.

[B013] Schuster RK, Sivakumar S (2017). The larval development of *Habronema muscae* (Nematoda: Habronematidae) affects its intermediate host, *Musca domestica* (Diptera: Muscidae). Parasitol Res.

[B014] Traversa D, Iorio R, Petrizzi L, De Amicis I, Brandt S, Meana A (2007). Molecular diagnosis of equid summer sores. Vet Parasitol.

[B015] Traversa D, Otranto D, Iorio R, Carluccio A, Contri A, Paoletti B (2008). Identification of the intermediate hosts of *Habronema microstoma* and *Habronema muscae* under field conditions. Med Vet Entomol.

[B016] Tyrnenopoulou P, Diakakis N, Psalla D, Traversa D, Papadopoulos E, Antonakakis M (2019). Successful surgical management of eosinophilic granuloma on the urethral process of a gelding associated with *Habronema* spp. infection. Equine Vet Educ.

[B017] Valentine BA (2005). Equine cutaneous non-neoplastic nodular and proliferative lesions in the Pacific Northwest. Vet Dermatol.

[B018] Verhaar N, Hermans H, van Rooij E, van Oldruitenborgh-Oosterbaan MS, Ensink J (2018). Case series: periocular habronemiasis in five horses in the Netherlands. Vet Rec.

[B019] Xiao L, Herd RP, Majewski GA (1994). Comparative efficacy of moxidectin and ivermectin against hypobiotic and encysted cyathostomes and other equine parasites. Vet Parasitol.

